# Effect of prior exposure to dopamine agonists on treatment with gabapentin enacarbil in adults with moderate-to-severe primary restless legs syndrome: pooled analyses from 3 randomized trials

**DOI:** 10.1186/s40734-015-0018-3

**Published:** 2015-03-30

**Authors:** William G Ondo, Neal Hermanowicz, Diego García Borreguero, Mark J Jaros, Richard Kim, Gwendoline Shang

**Affiliations:** University of Texas Health Science Center at Houston, 6410 Fannin Street, Suite 1010, Houston, TX 77030 USA; University of California Irvine Movement Disorders Program, 100 Irvine Hall, Irvine, CA 92697 USA; Sleep Research Institute, Alberto Alcocer 19, 28036 Madrid, Spain; Summit Analytical, LLC, 2422 Stout Street, Denver, CO 80205 USA; XenoPort, Inc., 3410 Central Expressway, Santa Clara, CA 95051 USA

**Keywords:** Restless legs syndrome, Gabapentin enacarbil, Dopamine agonist, International restless legs syndrome rating scale, Clinical global impression–improvement, Augmentation

## Abstract

**Background:**

Dopamine agonists (DAs) are a first-line therapy for moderate-to-severe restless legs syndrome (RLS), but these treatments may lead to complications, such as augmentation and impulse control disorders, requiring switching to another therapeutic class. Here we assess efficacy and tolerability of gabapentin enacarbil (GEn) in adults with moderate-to-severe primary RLS, with or without prior DA exposure.

**Methods:**

Data from 3 trials were pooled. Patients were identified as DA-naive or DA-exposed, based on prior treatment with ropinirole, pramipexole, rotigotine, or pergolide mesylate, and the dopamine precursor levodopa. Details on prior treatment duration and dose were unavailable. Patients with a history of augmentation were excluded. Within DA-naive/DA-exposed patients we investigated the co-primary end points from the pivotal trials: mean change from baseline to week 12 in International RLS (IRLS) Rating Scale total score and proportion of responders (“much”/“very much” improved) on the investigator-rated Clinical Global Impression–Improvement (CGI-I) scale. Safety was also assessed.

**Results:**

671 patients were randomized (DA-naive: placebo, n = 194; GEn 600 mg, n = 131; GEn 1200 mg, n = 214; DA-exposed: placebo, n = 50; GEn 600 mg, n = 30; GEn 1200 mg, n = 52). Across treatment arms, no significant differences between DA-naive and DA-exposed subgroups in IRLS Rating Scale total score change from baseline at any visit were seen, except week 1 in the placebo group (−6.1 DA-naive vs −3.4 DA-exposed, *P* = .020). No significant differences in the odds of CGI-I response at week 12 between DA-naive vs DA-exposed patients in any treatment group were seen; however, with placebo there was a nonsignificant trend toward fewer responders among DA-exposed (34.0%) vs DA-naive (44.3%) patients. Both GEn doses significantly improved the IRLS Rating Scale total score change from baseline and CGI-I response vs placebo, regardless of prior DA exposure. The most common treatment-emergent adverse events were dizziness and somnolence.

**Conclusions:**

Prior DA exposure had no significant effect on efficacy or tolerability of GEn (600 or 1200 mg) in this pooled analysis of adults with moderate-to-severe primary RLS. These data support the use of GEn in DA-exposed and DA-naive patients.

**Trial registration:**

ClinicalTrials.gov NCT00298623, NCT00365352, and NCT01332305

## Background

Restless legs syndrome (RLS), also known as Willis-Ekbom disease, is a common neurological disorder characterized by an urge to move the legs. This urge is frequently accompanied by unpleasant sensations in the legs, worsens in the evening and at rest, and is transiently improved with activity [1,2]. Patients with RLS experience significant impairments in sleep, daytime or social functioning, and overall quality of life [3,4].

Over the past decade, dopamine agonists (DAs) have been used as first-line therapy for patients with moderate-to-severe primary RLS [5]. Three DAs—ropinirole, pramipexole, and rotigotine—have been approved by the US Food and Drug Administration (FDA) for the treatment of moderate-to-severe primary RLS [2]. Though initially effective, the benefit of treatment with DAs may lessen over time owing to a variety of factors which may include augmentation, tolerance, or dopaminergic down-regulation. In particular, augmentation leads to a paradoxical scenario involving a worsening and earlier phase shift of RLS symptoms during treatment [6,7]. Some RLS patients who are treated with DAs may also develop impulse control disorders [2,8]. These developments may warrant switching to an alternate class of drugs when these side effects develop.

Gabapentin enacarbil (GEn), an agent in the alpha-2-delta ligand class of drugs, is an actively transported prodrug of gabapentin. GEn is approved by the FDA at a dose of 600 mg once daily for the treatment of moderate-to-severe primary RLS in adults. GEn is also approved for the management of postherpetic neuralgia in adults (600 mg twice daily) [9] and remains the only FDA-approved non-DA alternative for the treatment of moderate-to-severe primary RLS. In 3 randomized, double-blind, placebo-controlled studies in adult patients with moderate-to-severe primary RLS (XP052/XP053/XP081), GEn (600 mg and 1200 mg) significantly improved RLS symptoms compared with placebo, as assessed by the mean change from baseline in International RLS (IRLS) Rating Scale total score and the proportion of responders on the investigator-rated Clinical Global Impression–Improvement (CGI-I) scale at week 12. In all 3 studies, the most commonly reported adverse events were somnolence and dizziness [10-12].

When physicians consider how to treat patients with RLS, the potential effect of prior DA exposure on the efficacy of the new agent could be a factor. The effect of starting alternative agents, such as GEn, following exposure to DAs has not yet been examined. To investigate the effect of prior DA exposure on response to GEn treatment in adult patients with moderate-to-severe primary RLS, we compared outcomes for patients with and without prior DA exposure using pooled data from the XP052, XP053, and XP081 studies.

## Methods

### Study design and patients

The study designs and patient populations of the 3 primary studies (XP052, XP053, and XP081) have been published previously (ClinicalTrials.gov NCT00298623, NCT00365352, and NCT01332305) [10-12]. These were phase 2 or 3, double-blind, 12-week, placebo-controlled trials in adults with moderate-to-severe primary RLS, as defined by the IRLS Study Group diagnostic criteria [13].

For this analysis, data were pooled for each treatment from the XP052 (GEn 1200 mg and placebo) and XP053 (GEn 600 mg, GEn 1200 mg, and placebo) studies. Patients were grouped based on previous DA treatment status. Considering that augmentation was an exclusion criterion from study participation, the rate of any preceding augmentation was not rigorously assessed and thus remains unknown. In addition, the extent of treatment duration and dose of prior DA therapy were also not available. All subjects provided written informed consent prior to study participation. The primary studies were conducted in accordance with good clinical practice guidelines and the Declaration of Helsinki.

Co-primary end points from the pivotal trials, and investigated in the present analysis, were mean change in IRLS Rating Scale total score from baseline to week 12 [14], and the proportion of responders (“much” or “very much” improved) on the investigator-rated CGI-I scale [15] at week 12 for GEn 600 mg and GEn 1200 mg compared with placebo. Safety outcomes included treatment-emergent adverse events (TEAEs) and serious AEs.

### Statistical analyses

Efficacy analyses were performed on the modified intent-to-treat population (all patients in the safety population with a baseline and ≥1 post-baseline IRLS Rating Scale total score). Missing data were imputed using the last observation carried forward for analyses of the investigator-rated CGI-I data, and a mixed-effect model for repeated measures (MMRM) with observed cases (no imputation) was used for analysis of the IRLS Rating Scale total score. The main comparisons for all efficacy analyses were within treatments across DA status (by visit for IRLS Rating Scale total score).

For the change from baseline IRLS Rating Scale total scores, the effects of prior DA exposure were analyzed using MMRM with an unstructured covariance matrix, including fixed effects for treatment, visit, treatment-by-visit interaction, baseline IRLS Rating Scale total score, DA history (yes/no), DA history-by-treatment interaction, DA history-by-visit interaction, and DA history-by-treatment-by-visit interaction. For the percentage of CGI-I responders, the effect of prior DA exposure was analyzed using a logistic regression model with the following factors: treatment, DA history (yes/no), and DA history-by-treatment interaction.

## Results

### Patients

In this pooled analysis, 19.7% (132/671) of patients had been previously exposed to DAs. Ropinirole was the most frequently reported prior DA across the 3 treatment arms (Table 1). In general, a greater proportion of DA-exposed patients had severe RLS at baseline compared with DA-naive patients (IRLS Rating Scale total score ≥24; 59.9% vs 42.7%). In the DA-naive group, 16.7% (90/539) of patients received non-DA prior treatment for RLS. Although not FDA-approved for the treatment of primary moderate-to-severe primary RLS, these non-DA prior treatments included gabapentin, non-steroidal anti-inflammatory drugs, sleep aids, clonazapam, tramadol, cyclobenzaprine, and quinine (Table 1). The mean duration of RLS symptoms was 12.5 to 13.9 years in the DA-naive group and 16.1 to 17.9 years in the DA-exposed group. Of the DA-naive patients, 82.0% (159/194) in the placebo group, 84.7% (111/131) in the GEn 600-mg group, and 86.0% (184/214) in the GEn 1200-mg group completed their respective study. Among the DA-exposed patients, 82.0% (41/50) in the placebo group, 90.0% (27/30) in the GEn 600-mg group, and 86.5% (45/52) in the GEn 1200-mg group completed their respective study (Table 1).

**Table 1:** **Baseline characteristics of DA-naive and DA-exposed patients (mITT population)**

**Characteristic**	**DA-naive (n = 539)**			**DA-exposed (n = 132)**		
	**Placebo (n = 194)**	**GEn 600 mg**	**GEn 1200 mg (n = 131)**	**Placebo (n = 50)**	**GEn 600 mg (n = 30)**	**GEn 1200 mg (n = 52)**
Age at screening, mean years (SD)	48.9 (12.78)	47.2 (12.85)	49.8 (11.99)	50.5 (10.33)	52.4 (11.26)	54.2 (14.40)
Sex, n (%)						
Female	122 (63)	75 (57)	122 (57)	29 (58)	21 (70)	31 (60)
Male	72 (37)	56 (43)	92 (43)	21 (42)	9 (30)	21 (40)
Race, n (%)						
White/Caucasian	186 (96)	123 (94)	206 (96)	48 (96)	29 (97)	50 (96)
Mean baseline IRLS Rating Scale total score, points (SD)	22.5 (4.76)	23.2 (4.98)	22.9 (5.07)	25.2 (4.72)	24.0 (5.34)	24.6 (5.28)
Mean IRLS Rating Scale total score, n (%)						
<24 at baseline	116 (60)	67 (51)	126 (59)	18 (36)	14 (47)	21 (40)
≥24 at baseline	78 (40)	64 (49)	88 (41)	32 (64)	16 (53)	31 (60)
Duration of RLS symptoms, years						
Mean (SD)	13.1 (12.29)	12.5 (12.82)	13.9 (13.33)	17.0 (15.13)	17.9 (12.09)	16.1 (16.80)
Prior RLS treatment, n (%)						
Yes^a^	39 (20)^c^	20 (15)^c^	31 (14)^c^	50 (100)	30 (100)	52 (100)
Prior dopamine agonist treatment, n (%)						
Ropinirole	N/A	N/A	N/A	39 (78)	26 (87)	38 (73)
Pramipexole	N/A	N/A	N/A	9 (18)	5 (17)	10 (19)
Levodopa-carbidopa^b^	N/A	N/A	N/A	7 (14)	2 (7)	4 (8)
Rotigotine	N/A	N/A	N/A	2 (4)	2 (7)	2 (4)
Pergolide mesylate	N/A	N/A	N/A	1 (2)	0	0
Levodopa^b^	N/A	N/A	N/A	0	0	1 (2)

^a^Includes patients whose treatment terminated prior to the month before the start of study drug, and those who received treatment within the month of the start of study drug or within the previous month. ^b^Classified as dopaminergic agents. Patients with a past history of treatment with levodopa-carbidopa and levodopa were included in the DA-exposed group. ^c^Examples of non-DA prior treatment include: gabapentin, non-steroidal anti-inflammatory drugs, zolpidem, diphenhydramine HCL, clonazepam, diazepam, trazodone, tramadol, propoxyphene, cyclobenzaprine, and quinine. Please note, these treatments are not FDA approved treatments for primary moderate-to-severe RLS and list is not inclusive of all prior treatments reported.

DA, dopamine agonist; GEn, gabapentin enacarbil; IRLS, International Restless Legs Syndrome; mITT, modified intent-to-treat; N/A, not available; RLS, restless legs syndrome; SD, standard deviation.

### End points

**Figure 1:**
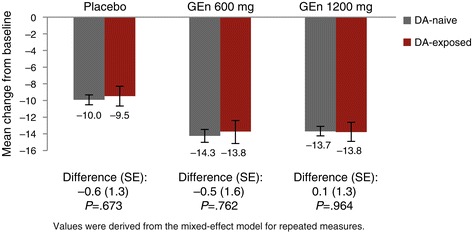
**IRLS Rating Scale total score change from baseline in DA-naive vs DA-exposed patients (week 12).** Change from baseline reported as the LS mean change from baseline. DA, dopamine agonist; Diff, mean treatment difference between DA-naive and DA-exposed treatment groups; GEn, gabapentin enacarbil; IRLS, International Restless Legs Syndrome; LS, least squares; SE, standard error.

**Figure 2:**
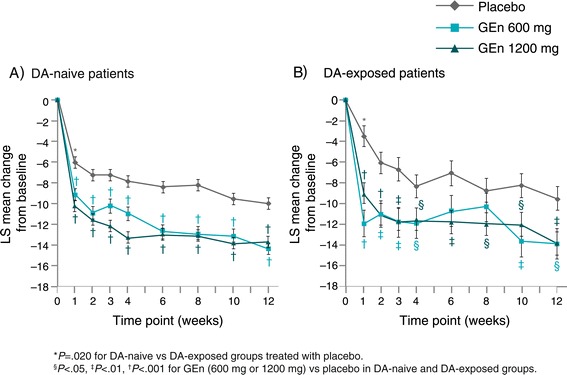
**IRLS Rating Scale total score changes from baseline by visit in DA-naive (A) and DA-exposed (B) patients.** MMRM analysis. Change from baseline reported as the LS mean change from baseline. Within the placebo group, there were no statistically significant differences in change in IRLS Rating Scale total score from baseline between the DA-naive vs DA-exposed patients in any treatment arm at any of the visits, except at week 1. Within the GEn 600-mg and GEn 1200-mg groups, there were no statistically significant differences in change in IRLS Rating Scale total score from baseline between the DA-naive vs DA-exposed patients in any treatment arm at any of the visits. DA, dopamine agonist; GEn, gabapentin enacarbil; IRLS, International Restless Legs Scale; LS, least squares; MMRM, mixed-effect model for repeated measures; W, week.

At week 12, prior DA exposure had no effect on the change in IRLS Rating Scale total score from baseline in the placebo (treatment difference between DA-naive and DA-exposed patients: −0.6 [1.3], *P* = .673), GEn 600-mg (treatment difference: −0.5 [1.6], *P* = .762), or GEn 1200-mg (treatment difference: 0.1 [1.3], *P* = .964) groups (Figure 1). With the exception of week 1 in the placebo group, there were no statistically significant differences in change in IRLS Rating Scale total score from baseline between the DA-naive vs DA-exposed patients in any treatment arm at any of the visits (Figure 2). At week 1, the change from baseline in the IRLS Rating Scale was greater in the DA-naive patients compared with the DA-exposed patients treated with placebo (−6.1 vs −3.4, *P* = .020).

**Figure 3:**
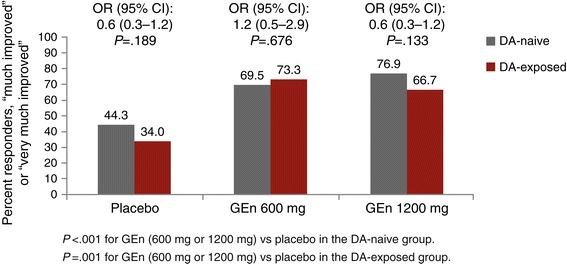
**Percentage of responders on the investigator-rated CGI-I in DA-naive vs DA-exposed patients (week 12).** Response on the CGI-I was defined as “much” or “very much” improved at week 12. CGI-I, Clinical Global Impression–Improvement; CI, confidence interval; DA, dopamine agonist; GEn, gabapentin enacarbil; OR, odds ratio.

There were also no significant differences in the odds of being a CGI-I responder between the DA-naive vs DA-exposed patients in any of the treatment groups at week 12 (Figure 3), although the DA-exposed placebo group showed a nonsignificant trend toward fewer responders compared with the DA-naive placebo group (34.0% vs 44.3%, respectively).

Regardless of whether they had prior DA exposure, patients showed significant improvements in IRLS Rating Scale total score change from baseline with both GEn doses compared with placebo at most time points (Figure 2). Significantly more patients receiving GEn (600 mg or 1200 mg) were CGI-I responders compared with patients receiving placebo in both DA-naive and DA-exposed groups (Figure 3).

### Tolerability

The TEAE profile was similar between DA-exposed and DA-naive patients. The most commonly reported TEAEs in the safety population were somnolence and dizziness (Table 2). Thirty-eight patients (7%) in the DA-naive group (GEn 600 mg, n = 10; GEn 1200 mg, n = 18; placebo, n = 10) and 6 patients (5%) in the DA-exposed group (GEn 600 mg, n = 1; GEn 1200 mg, n = 5; placebo, n = 0) discontinued treatment because of an AE. The majority of AEs were rated as mild or moderate in intensity. A total of 6 patients reported serious AEs: cellulitis (DA-naive, GEn 600 mg), worsened peripheral arterial disease (DA-naive, placebo), worsening cholelithiasis (DA-naive, GEn 1200 mg), cholelithiasis (DA-exposed, placebo), appendicitis (DA-exposed, placebo), and herniated disc (DA-exposed, GEn 600 mg). None of these events were considered treatment-related, and 5 of the 6 patients recovered (the outcome of 1 patient with worsened peripheral arterial disease was unknown).

**Table 2:** **Most frequent TEAEs in ≥5% of the safety population of any treatment groups** ^**a**^

**Adverse event, n (%)**	**DA-naive**			**DA-exposed**		
	**Placebo (n = 195)**	**GEn 600 mg (n = 133)**	**GEn 1200 mg (n = 217)**	**Placebo (n = 50)**	**GEn 600 mg (n = 30)**	**GEn 1200 mg (n = 52)**
Any event	149 (76)	105 (79)	187 (86)	34 (68)	27 (90)	40 (77)
Somnolence	12 (6)	25 (19)	54 (25)	0	7 (23)	7 (14)
Dizziness	9 (5)	20 (15)	44 (20)	2 (4)	2 (7)	15 (29)
Headache	22 (11)	12 (9)	36 (17)	5 (10)	7 (23)	4 (8)
Nasopharyngitis	10 (5)	13 (10)	17 (8)	6 (12)	1 (3)	4 (8)
Nausea	8 (4)	5 (4)	15 (7)	4 (8)	4 (13)	4 (8)
Fatigue	10 (5)	5 (4)	15 (7)	1 (2)	3 (10)	3 (6)
Diarrhea	10 (5)	3 (2)	7 (3)	2 (4)	3 (10)	3 (6)
Upper respiratory tract infection	7 (4)	6 (5)	5 (2)	2 (4)	3 (10)	1 (2)
Dry mouth	4 (2)	4 (3)	11 (5)	1 (2)	1 (3)	1 (2)
Constipation	6 (3)	0	7 (3)	2 (4)	3 (10)	3 (6)
Insomnia	6 (3)	7 (5)	4 (2)	1 (2)	1 (3)	2 (4)
Irritability	3 (2)	2 (2)	8 (4)	0	4 (13)	3 (6)
Back pain	6 (3)	4 (3)	6 (3)	1 (2)	2 (7)	1 (2)
Sinusitis	5 (3)	2 (2)	6 (3)	1 (2)	3 (10)	1 (2)
Increased weight	3 (2)	2 (2)	7 (3)	2 (4)	2 (7)	2 (4)

^a^Additional AEs reported in ≥5% of the safety population (at an overall frequency lower than those shown in the table) were: flatulence, contusion, abnormal coordination, toothache, increased appetite, urinary tract infection, depression, viral gastroenteritis, neck pain.

DA, dopamine agonist; GEn, gabapentin enacarbil; TEAE, treatment-emergent adverse events.

## Discussion

In this pooled analysis of 671 adult patients with moderate-to-severe RLS, previous exposure to DAs did not significantly alter the efficacy and TEAE profile of GEn (600 mg or 1200 mg) given once daily compared with placebo. There were no significant differences in the change in IRLS Rating Scale total score (looking at change from baseline both at week 12 and by visit) or in the investigator-rated CGI-I responder status between the DA-naive and DA-exposed patients in any of the treatment groups. Although these findings are preliminary, it is worth noting that the limitations of these analyses include the fact that information on prior DA treatment duration and dose were not available nor was the rate of augmentation rigorously assessed, particularly because patients with symptom augmentation were excluded from the trial. Regardless of prior DA exposure, both GEn doses significantly improved change from baseline in IRLS Rating Scale total score and CGI-I response compared with placebo. The TEAE profile was similar between DA-naive and DA-exposed patients, with somnolence and dizziness being the most commonly reported TEAEs. The TEAEs reported in this study are consistent with those of the primary analyses [10-12] and with the overall safety profile of GEn [9].

Although DAs are the most commonly prescribed agents for the treatment of RLS [5,7], there is mounting concern about AEs associated with treatment with DA, particularly augmentation and impulse control disorders [16]. Augmentation involves an increase in the duration (earlier onset), anatomy, and intensity of RLS symptoms. Further, the incidence and severity of augmentation increases linearly with the duration of DA treatment and usually resolves over weeks once DA treatment is discontinued [6]. This period, however, can be complicated by severe RLS symptoms, which are not always resolved by switching from one DA to another [17,18]. These are important points to note when considering treatment with DAs, as the dose and duration of prior DA exposure are related to the likelihood of augmentation, and augmentation is associated with a decrease in the response to subsequent DA treatment [16]. A retrospective analysis found that DAs are also associated with impulse control disorders including pathological gambling, compulsive shopping, and hypersexuality [19]. These data show the need for more prominent warnings for DAs as part of their prescribing information.

To our knowledge, the effect of starting non-DA treatments after a short washout period has not been studied in adequately controlled clinical trials, although a case report detailed that the alpha-2-delta ligand pregabalin improved RLS symptoms in a patient previously treated with the DA pramipexole [20]. According to recent guidelines, patients who experience augmentation due to DAs may benefit from alternative treatments, including alpha-2-delta ligands such as GEn [4], but no studies have investigated this. Our analysis suggests that GEn can be used to effectively treat RLS symptoms in patients with or without prior DA treatment, after a short washout period.

RLS studies can also be complicated by a robust placebo response, and prior DA treatment status has been shown to affect placebo response in patients with RLS [21,22]. In a meta-analysis of double-blind, randomized, placebo-controlled studies of patients with RLS, the placebo effect was particularly large for the primary outcome measure (IRLS Rating Scale) but less so for scales of daytime functioning [21]. Our analysis also found a treatment difference in the change in IRLS Rating Scale total score from baseline between DA-naive and DA-exposed populations within the placebo group at week 1. A blunted short-term placebo response resulting from prior DA treatment could be a possible explanation for these data.

Our analysis is limited to the data available from the individual trials. In particular, data on prior DA exposure is limited to the type of DA used, and the washout period following DA treatment was ≥2 weeks. As noted previously, information is not available to describe the duration, dosage, and other details of previous dopaminergic treatment, leaving an important gap. As the severity and duration of RLS symptoms were greater in the DA-exposed group than in the DA-naive group, one might expect the DA-exposed group to have less of a response to GEn treatment; however, this was not the case. Although DA treatment ended at least 2 weeks before study initiation, the exact washout period following DA treatment was also unknown, raising the question of potential additive effects of DA treatment in the DA-exposed group. In addition to these limitations, the sample size of the DA-naive group was considerably larger than the DA-exposed group, and there were fewer patients with severe RLS in the DA-naive group than in the DA-exposed group, as one might expect. This was not a formal meta-analysis; therefore, the scope of this pooled analysis was limited to the GEn doses, treatment duration, and patient populations assessed in the XP052, XP053, and XP081 trials. Despite these limitations, the question whether previous exposure to a DA affects the efficacy of an alternate agent after changing treatment remains important clinically, and further studies that address these limitations properly are necessary. In particular, it would be worthwhile to examine prospectively whether earlier vs more delayed treatment with a non-DA medication, such as GEn, may lead to differential treatment effects in patients with RLS.

## Conclusions

In summary, limited, prior exposure to DA had no significant effects on the efficacy or tolerability of GEn (600 mg or 1200 mg) once daily in this pooled analysis of adult patients with moderate-to-severe primary RLS. These preliminary data might support the use of GEn in non-augmented patients who were previously treated with DA after a ≥2-week washout period, as well as in those who are DA-naive.

## Abbreviations

CGI-I: Clinical Global Impression–Improvement
DA: Dopamine agonist
FDA: United States Food and Drug Administration
GEn: Gabapentin enacarbil
IRLS: International Restless Legs Syndrome
mITT: Modified intent-to-treat
MMRM: Mixed-effect model for repeated measures
RLS: Restless legs syndrome
TEAE: Treatment-emergent adverse event
